# A Mediterranean Diet to Improve Cardiovascular and Cognitive Health: Protocol for a Randomised Controlled Intervention Study

**DOI:** 10.3390/nu9020145

**Published:** 2017-02-16

**Authors:** Alexandra T. Wade, Courtney R. Davis, Kathryn A. Dyer, Jonathan M. Hodgson, Richard J. Woodman, Hannah A. D. Keage, Karen J. Murphy

**Affiliations:** 1 Alliance for Research in Exercise, Nutrition and Activity, School of Health Sciences, University of South Australia, GPO Box 2471, Adelaide SA 5001, Australia; alexandra.wade@mymail.unisa.edu.au (A.T.W.); courtney.davis@mymail.unisa.edu.au (C.R.D.); kate.dyer@unisa.edu.au (K.A.D.); 2 School of Medicine and Pharmacology, Faculty of Medicine, Dentistry and Health Sciences, University of Western Australia, 35 Stirling Highway, Crawley WA 6009, Australia; jonathan.hodgson@uwa.edu.au; 3 School of Medical and Health Sciences, Edith Cowan University, Joondalup WA 6027, Australia; 4 Flinders Centre for Epidemiology and Biostatistics, Flinders University, GPO Box 2100, Adelaide SA 5001, Australia; richard.woodman@flinders.edu.au; 5 Cognitive Ageing and Impairment Neurosciences, School of Psychology, Social Work and Social Policy, University of South Australia, GPO Box 2471, Adelaide SA 5001, Australia; hannah.keage@unisa.edu.au

**Keywords:** Mediterranean diet, cardiovascular, CVD, cognitive function, study protocol, randomised controlled trial

## Abstract

The Mediterranean diet has demonstrated efficacy for improving cardiovascular and cognitive health. However, a traditional Mediterranean diet delivers fewer serves of dairy and less dietary calcium than is currently recommended in Australia, which may limit long-term sustainability. The present study aims to evaluate whether a Mediterranean diet with adequate dairy and calcium can improve cardiovascular and cognitive function in an at-risk population, and thereby reduce risk of cardiovascular disease (CVD) and cognitive decline. A randomised, controlled, parallel, crossover design trial will compare a Mediterranean diet supplemented with dairy foods against a low-fat control diet. Forty participants with systolic blood pressure above 120 mmHg and at least two other risk factors of CVD will undertake each dietary intervention for eight weeks, with an eight-week washout period between interventions. Systolic blood pressure will be the primary measure of interest. Secondary outcomes will include measures of cardiometabolic health, dietary compliance, cognitive function, assessed using the Cambridge Neuropsychological Test Automated Battery (CANTAB), psychological well-being and dementia risk. This research will provide empirical evidence as to whether the Mediterranean diet can be modified to provide recommended dairy and calcium intakes while continuing to deliver positive effects for cardiovascular and cognitive health. The findings will hold relevance for the field of preventative healthcare and may contribute to revisions of national dietary guidelines.

## 1. Introduction

### 1.1. Background

Worldwide, cardiovascular disease (CVD) is the leading cause of death [1], while dementia is one of the greatest contributors to disability and dependence for individuals over the age of 60 [2]. Due to population growth and demographic ageing the prevalence and cost of age-related diseases and disorders, such as CVD and dementia, will continue to rise. Dementia currently affects 46.8 million people and numbers are expected to double every 20 years and reach 131.5 million by 2050. Between 2010 and 2015 the global cost of dementia increased by 35.4% to $818 billion USD, and is predicted to reach $2 trillion USD by 2030 [2]. Similarly, projections indicate that costs associated with CVD will triple by 2030 [3,4].

CVD and dementia share common risk factors including ageing, smoking, obesity, hypertension, dyslipidaemia and diabetes mellitus. Individually and collectively, these risk factors contribute to an inflammatory process that compromises endothelial function and promotes the development of atherosclerosis [5,6]. Atherosclerotic disease is the leading cause of CVD, coronary artery disease and cerebrovascular disease, and a significant predictor of late-life dementia and Alzheimer’s disease (AD) [7,8]. 

The same risk factors may further contribute to AD and dementia through independent pathways. Hypertension, for example, is capable of altering brain structure and volume, particularly in regions of the brain vulnerable to ageing and associated with AD, such as the prefrontal cortex [9,10,11]. Together, hypertension and atherosclerosis exacerbate the already reduced cerebral blood flow associated with normal ageing [12,13], increasing the incidence of hypoxia and neuritic plaque accumulation, effectively leading to cell degeneration and death [14]. 

To alleviate the social and economic burden of CVD, recommendations have called for health and lifestyle interventions targeting multiple risk factors [8]. Furthermore, recent calculations indicate that up to one third of AD can be attributed to modifiable risk factors (such as hypertension, obesity and diabetes), and reducing these will significantly reduce the prevalence of AD [15]. 

### 1.2. Dietary Intervention

Diet is a cornerstone treatment target for primary and secondary CVD prevention [16,17]. Dietary changes (such as replacing saturated with unsaturated fats; increasing fibre, fruit and vegetable intake; reducing salt and discretionary food intake; and restricting total energy intake) have been effective for the treatment of obesity, hypertension, hyperglycaemia and dyslipidaemia [18,19,20,21,22,23,24,25,26,27,28] and may improve cognitive function and well-being [29,30,31,32,33,34,35]. Further, specific nutrients in the diet may play a positive role in cardiovascular and cognitive health. For example, the consumption of polyphenols, flavonoids and antioxidants has been linked to improvements in blood pressure, endothelial function and reductions in pro-inflammatory markers associated with atherosclerosis, such as C-reactive protein (CRP) [36,37,38,39,40,41,42]. Regular consumption of flavanols and other polyphenols has also led to improvements across the cognitive domains of processing speed, memory and global cognition in healthy adults [43,44,45,46,47], and improved cognitive performance in older adults with mild cognitive impairment (MCI) [48]. 

Individually, these dietary modifications have demonstrated positive effects on cardiovascular and cognitive outcomes. However, an integrative, whole-of-diet approach may offer further benefit.

#### The Mediterranean Diet

The Mediterranean dietary pattern is characterised by high consumption of extra virgin olive oil (EVOO), vegetables, fruit, nuts, seeds, legumes and cereals; moderate consumption of fish, poultry, dairy and red wine; and lower consumption of eggs, red and processed meat and processed foods [49]. The primary foods of the Mediterranean diet contain bioactive nutrients and phytochemicals including mono- and poly-unsaturated fatty acids such as omega-3, polyphenols including flavonoids, vitamins, minerals and fibre, arginine and antioxidants. 

Cross-sectional and prospective studies indicate that populations with higher adherence to a Mediterranean diet exhibit lower risk of CVD and AD [50,51,52,53,54]. Intervention studies examining the Mediterranean diet have reported improvements to blood pressure, lipid profiles, insulin sensitivity, CRP levels and oxidative stress [37,41,55], and amelioration of atherosclerotic disease has been observed [56]. Further, a Mediterranean diet has been shown to improve function across cognitive domains affected by dementia and AD, even after short-term interventions [57,58]. Notably, improvements in cognitive functions sensitive to dementia and AD may translate to a reduced risk or delayed onset of dementia and AD [59,60]. 

The Prevención con Dieta Mediterránea (PREDIMED) study, a long-term large-scale randomised controlled trial, examined the efficacy of a Mediterranean diet against a low-fat diet in a Spanish population at risk of developing CVD [61]. After a median follow-up period of 4.8 years, risk of CVD and incidence of major cardiovascular events was significantly reduced in groups following a Mediterranean diet compared to those following a low-fat diet. Groups following a Mediterranean diet also demonstrated improved cognitive function [62,63] and rates of MCI, a risk factor and early indicator of dementia, were significantly lower than in control group [62]. 

Improved cognitive function following consumption of a Mediterranean diet may be the result of improved cardiovascular health. For instance, reduced inflammation and improved endothelial function encourage vasodilation and cerebral blood flow, reducing oxidative stress and neuronal cell death [64]. Alternatively, constituents of the Mediterranean diet may affect brain physiology directly, as individual nutrients have been shown to influence cognitive function in isolation [65,66,67,68,69,70]. For example, long-chain omega-3 fatty acids are integral to neuronal membrane functioning [71]. Similarly, flavonoids, polyphenolic compounds obtained from fruits and vegetables, tea and red wine, have been identified for their role in protecting against oxidative stress and neuronal death [72]. 

The potential for the Mediterranean diet to improve health outcomes is significant and has been acknowledged at the level of national nutritional policy. Recent modifications to the American Dietary Guidelines, for example, now include the Mediterranean diet as a healthy eating pattern [73,74].

### 1.3. Dairy Foods 

The Mediterranean diet shows promise for the prevention of CVD and dementia. However, before the diet is recommended as a long-term intervention it is necessary to ensure that it meets the nutritional requirements of the target population. 

Notably, a traditional Mediterranean diet does not satisfy current Australian guidelines for dairy foods. The Australian National Health and Medical Research Council recommend 2.5 to 4 serves of dairy foods, such as milk, cheese and yoghurt each day for adults (depending on age and gender) [75]. In contrast, a typical Mediterranean diet only provides 1–2 serves of dairy foods per day [49]. 

Dairy foods deliver a range of essential vitamins, minerals, peptides and micronutrients. These include calcium, iodine, vitamins A, B_6_ and B_12_, riboflavin, magnesium, potassium, phosphorus and zinc. Prior analysis indicates that the Mediterranean dietary pattern, as a whole, meets Australian requirements for most of these nutrients, with the exception of zinc (due to the complex nature of the bioavailability of zinc and estimating dietary requirements) and calcium [49].

Adequate dietary calcium is essential to skeletal function and integrity [76]. This is of particular relevance to an ageing population who are at a heightened risk of bone loss, bone fracture and osteoporosis [77,78]. Adequate calcium is also vital to cognitive function, as synaptic transmission and the neuronal processes involved in learning and memory are dependent on calcium signalling [79].

Calculations suggest that calcium intake on a Mediterranean diet is approximately 700–820 mg/day [80,81]. This meets the recommended dietary intake (RDI) of 750 mg for European countries [82] but is below both the estimated average requirement (EAR) (840–1100 mg/day) and the RDI for Australians, where women up to the age of 50 and men up to the age of 70 are advised to consume 1000 mg daily, and 1300 mg thereafter [76]. Recommended daily intakes are indicative of the traditional intakes of a population, accounting for deficits and needs. The difference between Australian and European RDIs for calcium may then be a reflection of the prevalence of osteoporosis. In Australia, it is estimated that 27% of men and 44% of women over the age of 50 will develop osteoporosis [83]. In contrast, Mediterranean countries such as Greece, Italy and Spain report a lower incidence of osteoporosis: prevalence over the age of 50 ranges from 6.8%–7% for men and 22%–23.5% for women [84]. 

For the long-term sustainability of a Mediterranean diet in an Australian population it is necessary to ensure that calcium requirements are met, especially in consideration of bone health concerns. Recommending a calcium intake of approximately 700–820 mg/day, as obtained from a traditional Mediterranean diet, might not be optimal or viable for an Australian population in whom a greater calcium intake still results in higher prevalence of osteoporosis, and where habitual calcium intake of less than 751 mg/day is associated with increased risk of osteoporosis [85]. 

While the impact of dairy foods in a Mediterranean diet is currently unknown, nutrients contained in dairy foods have been identified for their potential to improve cardiovascular risk factors. For example, calcium has been linked to weight loss and improvements in lipid profiles and insulin sensitivity [86,87,88]; magnesium may be beneficial for blood pressure, insulin sensitivity and lipid profiles [88,89,90]; and phosphorus from dairy foods has been linked to reduced risk of hypertension [91]. Moreover, dairy food consumption is associated with better outcomes for blood pressure, inflammation, insulin sensitivity, and CVD [41,92,93,94,95,96]. By improving cardiovascular health, dairy foods may also be capable of improving cognitive function. Further, constituents of dairy foods, such as bioactive peptides, amino acids and vitamin B_12_ may affect brain physiology to improve cognitive function directly [97]. Despite this, clinical trials have reported both positive and negative relationships between dairy intake and cognitive functioning [98,99,100]. The potential impact of added dairy on cognition therefore requires further attention.

Increasing serves of dairy foods in the Mediterranean diet, to supply adequate calcium for an Australian population may increase endorsement, adoption and sustainability for an Australian population. However, foods are not consumed in isolation, but are instead components of a dietary pattern. It is therefore unclear whether additional dairy will compromise or work in synergy with other elements of the Mediterranean diet. 

### 1.4. Objectives

The current study aims to evaluate the effect of a Mediterranean diet that meets Australian dairy and calcium recommendations. Specifically, a Mediterranean diet supplemented with additional dairy (milk, cheese and yoghurt) will be compared with a low-fat control diet on measures of cardiovascular risk and cognitive performance, quality of life and psychological well-being. A secondary aim of the current study is to determine the extent to which intervention effects on cognitive function are dependent on improvements to cardiovascular health.

For the primary prevention of CVD, targeting individuals at high risk may be more effective than targeting those at low or medium risk [101]. To evaluate the potential of a Mediterranean diet to reduce risk, the intervention will be administered to a sample with multiple risk factors of CVD, who are therefore at high risk of CVD and premature cognitive decline. As blood pressure a modifiable and strong predictor of CVD, brain atrophy and dementia, home blood pressure will be used as the primary outcome measure. Secondary outcomes will include measures of cardiometabolic health such as diastolic blood pressure, body mass index (BMI), waist-to-hip ratio, body composition, red blood cell fatty acids, blood lipids, Apolipoprotein E4 (ApoE4), C-reactive protein (CRP), blood glucose and insulin, estimated risk of dementia and measures of cognitive function. Specifically, memory, processing speed and executive function will be examined as each of these is sensitive to age-related decline, and task performance is predictive of cognitive ageing and risk of dementia and Alzheimer’s disease [30,102,103,104,105,106,107,108]. Further, these cognitive functions are negatively affected by hypertension, endothelial dysfunction, atherosclerosis and CVD [10,103,104,109,110,111,112,113,114,115,116,117,118,119].

The findings of this research will provide insight as to whether a Mediterranean diet that meets the dairy food group and calcium requirements of the target population is still capable of reducing risk of CVD and cognitive decline.

## 2. Materials and Methods 

### 2.1. Ethics

Ethical approval has been obtained from the University of South Australia Human Ethics Committee. The trial was registered with the Australia and New Zealand Clinical Trials Registry (ACTRN12616000309482) on 9 March 2016.

### 2.2. Participants 

#### 2.2.1. Inclusion Criteria

Volunteers aged between 45 and 75 with elevated systolic blood pressure above 120 mmHg and who are not taking antihypertensive medication will be recruited. To be eligible, volunteers will have at least two other risk factors for CVD, including: a body mass index ≥25 kg/m^2^; abdominal adiposity (waist circumference >94 cm for men and >80 cm for women); elevated total cholesterol (≥5.5 mM), triglycerides (≥2.0 mM), low-density lipoprotein (LDL) (≥3.5), or low levels of high-density lipoprotein (HDL) (≤0.9 for men and ≤1.0 for women); impaired glucose tolerance (between 6.1 and 7.8 mmol/L); and/or a family history of CVD or type 2 diabetes. 

#### 2.2.2. Exclusion Criteria 

Current smokers, individuals with medical conditions including: current CVD or angina, current or recent malignancies, kidney disease, gastrointestinal disease, respiratory disease, Type 2 diabetes mellitus, a current or previous traumatic head or brain injury, a current neurological or psychiatric condition, or a current diagnosis of Alzheimer’s disease or dementia will be excluded from participation. Volunteers with dietary requirements, aversions, allergies or intolerances, such as fish and nut allergies or lactose intolerance, that will interfere with their ability to follow dietary guidelines will not be invited to participate. Further, individuals taking medicinal levels of calcium or >1000 mg of omega-3 supplements daily will not be eligible. 

#### 2.2.3. Recruitment and Screening

Volunteers will be recruited through electronic and paper media advertisements and assessed for eligibility through a diet and lifestyle questionnaire (DLQ) and screening visit at the Sansom Institute Clinical Trial Facility. The DLQ will include questions regarding medical history, medications and supplements, family medical history, dietary requirements, aversions, allergies and intolerances. At the screening visit the Addenbrooke’s Cognitive Exam-Revised (ACE-R) [120] will be administered to detect pre-existing MCI and dementia. Blood pressure will be measured as detailed below, in addition to anthropometry (height, weight, waist and hip measurements), fasting blood glucose using a finger-prick glucose test. A fasting blood sample will also be collected to measure blood lipid profile.

### 2.3. Design

**Figure 1:**
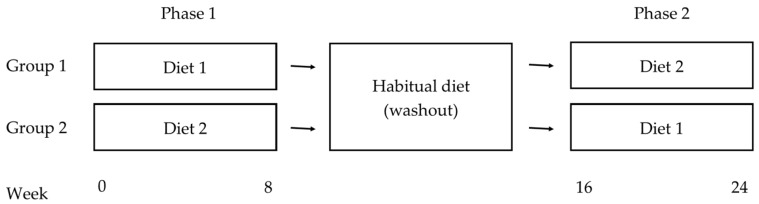
Crossover design.

The current study will employ a 24-week 2 × 2 crossover design. Participants will complete two 8-week dietary interventions, separated by an 8-week washout period. Participants will be allocated to a starting diet group, either the Mediterranean diet or the low-fat diet, using block randomisation and stratification based on age and gender. In Phase 1, participants will follow their starting diet for 8 weeks. All participants will then return to their habitual diet for 8 weeks, after which they will “cross-over” and complete the second 8-week intervention for Phase 2 (see Figure 1). All participants will therefore complete each experimental condition. 

The crossover design has been chosen for time and resource efficiency. As participants will act as their own control, potential differences between subjects in both observed and unobserved confounders that may be present in a medium-sized parallel-group design will be removed. This will reduce the potential for bias in estimating the treatment effect. In addition, repeat measures on the same individual allows for separate estimation of the true underlying value for each individual, the treatment effect and the measurement error. Separating the treatment effect from the measurement error will allow a more precise estimation of the treatment effect and fewer participants will therefore be required to detect any between-group differences. The reduced bias due to the removal of potential confounding variables will also enhance the internal validity of the design. An 8-week intervention period is expected to be adequate as similarly timed RCTs have demonstrated significant effects of nutrients contained in the Mediterranean diet, such as flavanols and omega-3s, on cardiovascular outcomes [46,48,121]. Further, the observed changes in cardiovascular outcomes were found to mediate improvements in cognitive function. As there is the potential for latency effects beyond 8 weeks, Potential carry-over effects will be assessed by testing for treatment × phase interactions, and it is anticipated that 8 weeks will be an adequate washout period to prevent cross-contamination between intervention phases [109]. Practice effects of cognitive tasks will be mitigated through the use of measures with strong test-retest reliabilities, counterbalancing of test order and the use of multi-level analysis that will allow the separation of measurement error (i.e., within-subject variability) from treatment effects.

### 2.4. Dietary Interventions

Weight loss has been linked to significant reductions in systolic and diastolic blood pressure, blood glucose, triglycerides and total cholesterol and inflammatory markers [122,123], as well as improvements to cognitive function [124], and so has the potential to mask the effects of a nuanced dietary intervention. To measure the effects of the intervention independent of weight loss, energy will not be restricted. Instead, participants will be instructed to consume all dietary interventions ad-libitum and self-regulate their food intake. If weight loss occurs the volunteer will be counselled to ensure that they are eating until satiated and that all dietary serving requirements are met. If weight loss continues changes in weight within each phase will be adjusted for statistically.

#### 2.4.1. The Low-Fat Diet (LFD)

Modelled on the PREDIMED study, participants will follow their habitual diet but will be instructed to reduce their total fat intake by restricting or avoiding intakes of high fat foods including all oil types, butter, margarine, full fat dairy, processed and high fat meats, cream, nuts, chocolate, cakes, biscuits, pastry and ice cream. Participants will be instructed to replace these foods with low fat alternatives such as breads, cereals, lean meats, legumes, rice, vegetables and fruits, or by choosing low fat variations (such as low fat dairy). Participants will be advised to consume no more than 20 mL of oil, no more than two teaspoons butter or margarine per day, and to remove visible fat and skin from meat and fish before cooking. As the LFD is intended to match participants’ habitual dietary intakes, recommendations will not be set for dairy consumption.

#### 2.4.2. The Mediterranean Diet Supplemented with Dairy (MedD)

While following the MedD intervention, participants will be instructed to adhere to the following guidelines (adapted from Estruch, Ros, Salas-Salvadó, Covas, Corella, Arós, Gómez-Gracia, Ruiz-Gutiérrez, Fiol, Lapetra, Lamuela-Raventos, Serra-Majem, Pintó, Basora, Muñoz, Sorlí, Martínez and Martínez-González [61] for Australian food supply):

- 3–4 daily servings of dairy foods (one serve = 250 mL low fat milk, 40–120 g hard and/or semisoft to soft cheese, 200 g low fat Greek yoghurt, or 200 g tzatziki dip);
- No more than one serving of cheese (any type) per day (one serve = 40 g hard, 50 g semi-soft, or 120 g soft cheese);
- Minimum of one tablespoon (20 mL) of EVOO per day;
- ≥2–3 daily servings of fresh fruit (one serve = 150 g fresh, 40 g dried, or one cup canned in juice);
- ≥3 weekly servings of legumes (one serve = 75 g);
- ≥3 weekly servings of fish and seafood (at least one serving of oily fish) (one serve = 100 g cooked);
- ≥3 weekly serving of nuts or seeds (one serve = 30 g; 7.5 g hazelnuts, 15 g walnuts, 7.5 g almonds supplied for each serve);
- Ad-libitum consumption of wholegrain cereal products (bread, pasta, rice, cereal), nuts, fish, eggs and raw and cooked vegetables;
- Select white meats (poultry without skin) instead of red meats or processed meats;
- Limit consumption of red meat (remove all visible fat), cured ham and chocolate to ≤1 serve/week (one serve of red meat/cured ham = 100 g; one serve of chocolate = 50 g);
- Use EVOO for cooking and dressing vegetables and salad;
- Cook regularly (at least twice a week) with a tomato based sauce (EVOO, tomato, garlic and onion);
- Dress vegetables, pasta, rice and other dishes with EVOO, tomato, garlic and onion sauce;
- Eliminate or limit the consumption of cream, butter, margarine, cold meat, pate, duck, carbonated and or sugared beverages, pastries, commercial bakery products (cakes, donuts, cookies), desserts (puddings), French fries, potato crisps, sweets;
- For usual drinkers, red wine is recommended as the main source of alcohol with a maximum of two standard drinks per day (200 mL = two standard drinks) [125].

Consistent with Australian Dietary Guidelines [75], serving sizes of dairy foods will deliver 300 mg of calcium. Three to four servings of dairy each day will therefore provide approximately 900 to 1200 mg of calcium, while vegetables and nuts will provide approximately 100 mg. Based on this modelling, the MedD diet will provide 1000–1300 mg of calcium each day, and thus meet the Australian RDI for calcium.

Although recommended for the control of blood pressure, restriction of dietary sodium is not a component of the Mediterranean diet and thus participants will not be instructed to reduce salt intake. 

To assist with adherence, the following foods will be provided: Chobani Greek yoghurt (donated by Chobani Australia Pty Ltd., Dandenong South, Victoria, Australia), almonds (donated by the Almond Board of Australia, Loxton, Australia), walnuts and hazelnuts, EVOO (donated by Cobram Estate, Southbank, Victoria, Australia), Mainland Tasty regular fat and reduced fat cheese slices (donated by Fonterra Co-Operative Group, Darnum, Victoria, Australi), Edgell chickpeas, cannellini beans, red kidney beans, 4-bean mix and lentils (donated by Simplot Australia Pty Ltd., Rose Park, Australia) and canned John West tuna “tempters” and salmon “tempters” (donated by Simplot Australia Pty Ltd.). 

Adverse effects of the Mediterranean diet may include constipation, diarrhoea and bloating due to a high intake of fibre. Participants will be encouraged to discuss any adverse effects during dietetic counselling sessions, in which the dietitian will offer advice to minimise discomfort. 

#### 2.4.3. Dietetic Counselling

At the beginning of each dietary phase, participants will meet with a dietitian for 45 min to discuss dietary guidelines. For the MedD phase, participants will be provided with a set of MedD diet guidelines (Appendix A), including serving sizes, guidelines to help incorporate dairy foods into their diet (Appendix B), education on serving sizes of various foods, and a Mediterranean diet recipe book. A weekly semi-quantitative checklist based on that employed in the Medley trial [126] will also be provided to assess adherence and to assist participants in becoming familiar with foods and amounts associated with a MedD diet (Appendix C). The checklist will be filled-in daily, using a tick system (1 tick = 1 serve consumed) and returned at each fortnightly visit. The dietitian will use this information as a discussion point, to assist the participant in following the diet. 

A similar approach will be used to educate participants in following the LFD. Each participant will be provided with a set of guidelines (Appendix D), education on fat content and portion sizes of various foods and a label reading handout (Appendix E), to assist with identifying fat content, including saturated and trans-unsaturated fat, when selecting packaged foods. 

All participants will attend fortnightly appointments throughout the intervention phases to meet with the dietitian and discuss their progress. Participants will be weighed to determine body mass. Should there be any gain, participants will be educated on their dietary choices, satiety, mindful eating and portion sizes. Any difficulties or adverse effects of the diet will be discussed, and strategies will be developed to assist in increasing adherence. Goal setting for the next fortnight, led by the participant with encouragement from the dietitian, will be conducted using the S.M.A.R.T goals for achieving high adherence (S, specific; M, measurable; A, achievable; R, realistic; T, time based).

### 2.5. Outcomes

#### 2.5.1. Blood Pressure

Home measured systolic blood pressure will be the primary outcome. Home blood pressure assessments have stronger predictive power for complications of hypertension and mortality than clinic blood pressure assessments [127,128]. Further, multiple home blood pressure measurements have comparable reliability to ambulatory blood pressure measurements in evaluating hypertension [129]. Participants will be provided with an A&D Company Ltd. digital blood pressure monitor (model UA-767) and instructed to measure their blood pressure and heart rate every morning, afternoon and evening, for a period of six days at each of the four study time-points. Participants will be asked to take measurements at consistent times each day, at least one hour after the consumption of caffeine or alcohol and 30 min after food or exercise. Each measurement of blood pressure will follow a five-minute resting period in the seated position and three readings will be taken, spaced at least one minute apart [130,131]. Home blood pressure will be recorded at four time-points over the course of the trial, one week prior to clinic assessment visits (at pre-baseline, Week 7, Week 15 and Week 23). Blood pressure will also be measured in-clinic using an Omron Healthcare Co. digital blood pressure monitor (model 1A1B Hem-7000-CIL) at the beginning and end of each dietary phase. A similar protocol for in-clinic measurements will be used, with blood pressure and heart rate measurements following a five-minute resting period in the seated position. Three readings will be taken at each visit, spaced at least one minute apart.

#### 2.5.2. Secondary Outcomes

Secondary cardiovascular outcomes will include diastolic blood pressure and heart rate, body mass index (BMI), waist-to-hip ratio, body composition (percentage body fat, lean mass and abdominal adiposity) assessed using dual-energy x-ray absorptiometry (DEXA), fasting blood lipids, CRP, fasting plasma glucose, fasting serum insulin, erythrocyte cell fatty acids and Apolipoprotein E4 (APOEε4). A faecal sample will also be collected to explore the effects of a Mediterranean diet with adequate calcium on gut microbiota.

Fasting venous blood will be collected by venepuncture and centrifuged (4 °C, 4000 rpm, 10 min) before plasma and erythrocytes are separately aliquoted and frozen at −20 °C and then stored at −80 °C. Lipids, CRP, glucose and insulin will be measured at an external NATA accredited laboratory using standard procedures. Insulin resistance will be calculated using The Homeostasis Model Assessment (HOMA2) Calculator v2.2.3 (University of Oxford, Oxford, UK) [132]. This method to determine an insulin resistance score (HOMA2-IR) has been validated in samples with varying degrees of insulin sensitivity, with no differences shown between gender, age, BMI or diabetes status [133]. A higher HOMA2-IR score indicates greater insulin resistance. Participants with fasting insulin levels >57 mU/L, indicating diabetes related metabolic disturbance, will be excluded from these calculations. Erythrocyte fatty acids will be measured using direct transesterification as described by Tu et al. (2013) [134]. APOEε4, an indicator of increased risk for AD, will be determined using the TaqMan^®^ SNP Genotyping assay kit (Applied Biosystems, Warrington, UK) [135].

##### Cognitive Performance, Dementia Risk and Mood

The cognitive domains of interest are verbal and visual memory, processing speed and executive function due to their demonstrated sensitivity to ageing and cardiovascular health, and their known responsiveness to short term nutritional interventions [68,106,136,137,138,139].

A set of cognitive tests from the Cambridge Neuropsychological Test Automated Battery (CANTAB) [140] will be administered to evaluate cognitive function across these domains. The selected tests include Motor Orientation Task (MOT), Paired Associates Learning (PAL), Delayed Matching to Sample (DMS), Verbal Recognition Memory (VRM), Reaction Time (RTI), Rapid Visual Information Processing (RVIP), Spatial Working Memory (SWM), Stockings of Cambridge (SOC) and Attention Switching Task (AST). A description of these tests and the cognitive functions examined by each is presented in Table 1.

The CANTAB offers parallel versions of each test for repeated measures designs to limit effects of learning. The test-retest reliability (*r*) of the chosen tests range from an *r* = 0.54 (RTI) to *r* = 0.87 (PAL) [141]. While test-retest coefficient less than *r* = 0.7 may compromise comparisons between repeated testing [142] the difference between real effects and learning effects can be determined by using standardised scores and their standard errors at each visit [141].

Addenbrooke’s Cognitive Exam-Revised (ACE-R) will be administered to screen for MCI and dementia and to detect change in cognitive function over the course of the trial. The ACE-R assesses attention, orientation, memory, fluency, language and visuospatial abilities and has demonstrated high sensitivity and specificity for detecting both MCI and dementia [120].

Dementia risk will be assessed through the Cardiovascular Risk Factors, Aging and Dementia (CAIDE) score and the Framingham vascular risk score. The CAIDE was developed to predict a 20-year risk of developing late-life dementia from middle age risk profiles [59] and is calculated on the basis of age, sex, education, hypercholesterolemia, systolic blood pressure, physical activity, obesity and apolipoprotein E genotype. The CAIDE has demonstrated high sensitivity (0.81) and moderate specificity (0.61). The Framingham vascular risk score calculates the risk of developing vascular disease based on age, sex, systolic blood pressure, hypertension, HDL cholesterol, total cholesterol, smoking and diabetes [143]. Notably, a higher cardiovascular risk score on the Framingham has been associated with greater declines across cognitive domains [144,145]. Compared to the CAIDE, the Framingham risk score has shown stronger associations with 10-year trajectories of cognitive decline [145].

Psychological well-being will be evaluated through the SF-36 Health Survey [146]. The SF-36 was designed to measure perceived health status through the constructs of vitality, physical functioning, bodily pain, general health perceptions, physical role functioning, emotional role functioning, social role functioning and mental health, and has reported sensitivity to change, high internal consistency, construct validity and adequate test-retest reliability [146]. Further, the SF-36 has demonstrated sensitivity to nutritional interventions [147,148,149]. To measure transient and enduring mood states, the Profile of Mood States (POMS) questionnaire will be employed. The POMS examines the constructs of tension-anxiety, depression-dejection, anger-hostility, vigour-activity, fatigue-inertia, and confusion-bewilderment. The POMS has demonstrated good internal consistency (Cronbach’s alpha = 0.63 to 0.96) and test re-test reliability of *r* = 0.61 to *r* = 0.69 at 6 weeks [150].

##### Diet Adherence

Adherence to the MedD intervention will be assessed using the weekly checklist (Appendix C, described above). Weekly totals of each food group will be compared against dietary guidelines to determine % adherence to the MedD diet. A 14-item Mediterranean diet adherence tool, adapted from the PREDIMED study to align with the Australian food supply and The Australian National Health and Medical Research Council (NHMRC) guidelines for alcohol consumption, will also be completed and returned at each fortnightly visit [61] (Appendix F). Adherence to the LFD will be measured using a 9-item low-fat diet adherence tool adapted from the PREDIMED study, completed and returned at each fortnightly visit [61] (Appendix G). These tools are designed to capture a generalised pattern of consumption relevant to the dietary phase. Finally, a 6-day weighed food record (WFR) will be completed before and during each dietary phase to calculate energy and macro and micronutrients intakes. 

Adherence to the whole dietary phase and the dairy food component of the MedD intervention will be determined at the first fortnight dietetic visit. Participants must meet a minimum of 75% adherence (as determined by checklists) to the relevant dietary prescription and a minimum of 75% adherence to the dairy recommendation (i.e., participants need to consume at least 3 serves dairy products per day to be compliant). If participants are unable to increase adherence above 75% in the next fortnight they will be excluded from the study.

In addition to the 14-point Mediterranean diet adherence tool, information from the 6-day WFR at the beginning and end of each dietary phase will be used to determine foods relevant to the Mediterranean diet, which will be selected and grouped appropriately to calculate adherence to a Mediterranean diet. Adherence will be determined through a modified 15-point score as described by Davis, Bryan, Hodgson and Murphy [49], based on a scoring system described by Trichopoulou, Costacou, Bamia and Dimitrios [54]. Relevant foods will be grouped into; vegetables, fruits, nuts, legumes, fish, breads, cereals, dairy and olive oil. For each of these groups, intakes (as grams/MJ) above the baseline group mean are awarded 1 point. Additional groups for sugars, eggs, potato, meat and miscellaneous are awarded 1 point for intakes below the baseline group mean. Previously the dairy food group was scored negatively (1 point for intakes below the mean) but will now be scored positively (1 point for intakes of 3 serves or more per day). For red wine, intakes of 200 mL per 8.7 MJ or below are awarded 1 point. 

### 2.6. Procedure 

Participants will attend two pre-baseline visits, four clinic assessment visits and six fortnightly visits at the Sansom Institute Clinical Trial Facility. 

Pre-baseline visits will take place one week before each dietary intervention begins. Participants will be provided with information about the study and asked to complete the SF-36, POMS, a six-day WFR and record home blood pressure for six days. The same measures will also be completed in the final week of each intervention at Weeks 7 and 23. 

Clinic assessment visits will take place at Weeks 0, 8, 16 and 24 (See Figure 1) and will include blood pressure measurement, anthropometry, DEXA scan and collection of fasted blood samples. Participants will be instructed to maintain their habitual levels of physical activity over the course of the trial. At each clinic assessment visit participants will be asked to provide details of habitual physical activity undertaken over the previous 2 months, including type of activity, frequency, duration and intensity. At Weeks 0 and 16 participants will be assigned their randomly allocated diet and will meet with the dietitian to discuss dietary guidelines. Participants will then consume a standardised continental breakfast before cognitive measures are administered. 

Fortnightly visits will be conducted throughout the intervention periods. At these visits weight will be measured and participants will have an opportunity to discuss their progress, challenges and goals with the dietician. 

The washout period will take place between the Week 8 and Week 16 clinic assessment visits, separating the two dietary intervention phases. 

Any changes to medications or dietary supplements will be noted throughout the course of the study and factored into the final analysis.

#### Cognitive Measures

A computerised mode of cognitive testing has been chosen to increase accuracy and reliability in detecting change, especially in instrument sensitive tasks such as reaction time [151]. To limit the influence of potential measurement bias, the administration of cognitive measures will be controlled and standardised across participants and visits, as described by Stonehouse et al. [152]. Cognitive assessments at Weeks 0, 8, 16 and 24 will be conducted at a similar time of day, between 9:30 a.m. and 12:00 p.m. Participants will have completed a 12-h fast from food, beverages (excluding water), caffeine and alcohol before arriving at the clinical trial facility. Following cardiometabolic assessments, anthropometry and DEXA scan, the same continental breakfast will be provided and eaten within 15 min of cognitive testing. If participants have not had an adequate amount or quality of sleep the preceding night cognitive testing will be rescheduled. The testing environment will be controlled with regard to temperature and noise in order to reduce distraction. A CANTAB familiarisation task will be performed at each testing session, and each cognitive task will have a practice component. Further, CANTAB administration will be standardised through use of a testing script provided by Cambridge Cognition.

### 2.7. Power Calculation and Statistical Analyses

In order to detect a clinically relevant difference of 2.5 mm Hg in the primary outcome measure of systolic home blood pressure with at least 90% power, a sample size of 31 volunteers is required. A reduction of 2.5 mm Hg in blood pressure translates to a risk reduction at a population level in stroke, hypertension and CVD of between 7% and 15% [133]. This calculation assumes a within-group standard deviation (SD) of 14 mmHg, a within-subject correlation between the 4 blood pressure measures at each visit of *r* = 0.6 based on previous studies [135,136], and a between-phase within-subject correlation of *ρ* = 0.5. This correlation (*ρ*) and the cross-over design reduces the number of required participants by a factor of (1 − *ρ*)/2 = 4 [153] i.e., from approximately *n* = 124 for a parallel group design using ANCOVA (*n* = 62 per group) to *n* = 31 subjects in total. To account for a withdrawal rate of 30%, an additional 9 volunteers will be recruited. 

To detect a statistically significant difference in *Z* scores for the cognitive outcomes Total Cognitive Functioning, Memory, Processing Speed and Executive Functioning, a sample size of *n* = 22 is required. This calculation assumes power equal to 0.8, an α level of 0.05, a correlation between measurements of 0.6, and a moderate effect size (Cohen’s *d* = 0.5) signifying a treatment effect of 0.5 SD [137]. A moderate effect size has been chosen on the basis of similar nutritional intervention studies that have detected significant improvements in cognitive outcomes, which may indicate restoration of cognitive function and reversal of cognitive decline [46,152].

Independent *t*-tests and chi-squared tests will be used to compare baseline demographic, cardiometabolic, cognitive, well-being and dietary characteristics between those who completed the study and those who withdrew.

To analyse the difference between dietary interventions a linear mixed-effects analysis will be performed to measure both within and between subject effects for cardiometabolic and cognitive outcomes. Potential confounding variables including weight loss, changes to physical activity, medications or dietary supplements, carryover and period effects will be included in the analysis. Potential carryover effects and treatment-period effects will be assessed by adding treatment × phase and treatment × order interaction terms in the mixed effects analyses. We will also assess possible seasonal effects by including a term for treatment × season interaction in a secondary analysis with season coded as a binary variable for Autumn or Spring versus Winter or Summer. Diet × period × energy intake interaction terms will be used to determine whether there are any significant changes in diet (total energy intake, macronutrient intakea, micronutrient intakes including sodium, and fatty acid ratios) between the two diet phases. If a significant change is detected in cognitive function a hierarchical regression will be performed to determine the influence of cardiometabolic outcomes. All statistical analyses will be conducted using SPSS for Windows, version 21.0 (SPSS Inc., Chicago, IL, USA) and Stata (version 14.2, StataCorp, College Station, TX, USA). Data will be presented as means ± standard deviation (SD) for descriptive statistics and as means ± standard error (SEM) for reporting estimated effects. All tests will be 2-tailed and *p*-values < 0.05 will be deemed statistically significant.

## 3. Discussion

Due to population ageing, the prevalence of illnesses such as CVD and dementia will continue to rise and the weight of their burden will be felt globally. Estimates suggest that delaying the onset of dementia by five years may reduce cost and prevalence by up to 44% [154,155]. Prophylactic measures endeavouring to reduce or delay the incidence of CVD and dementia therefore warrant thorough attention. 

Randomised controlled trials and cross-sectional investigations suggest that the Mediterranean diet may be effective for preventing both CVD and dementia. However, the Mediterranean diet is typically lower in dairy foods and calcium than Australian recommendations and long-term adherence may compromise health across the lifespan. The current paper outlines the protocol for a randomised controlled trial that will evaluate the impact of increased dairy intake in a Mediterranean dietary pattern to meet Australian recommendations for dairy foods and calcium. A comprehensive set of cardiovascular and cognitive outcomes will be measured to determine whether the Mediterranean diet with added dairy can significantly reduce CVD and dementia risk.

## 4. Conclusions

The findings of this research will have significant relevance to the field of preventative health care in offering empirical, evidence-based support for a potential preventative approach to promoting cardiovascular and cognitive health and reducing the risk and impact of disease.

## Figures and Tables

**Table 1:** Description of CANTAB tests and cognitive functions measured.

Test	Description	Cognitive Function
Motor Orientation Task (MOT)	Familiarisation task. The participant is instructed to touch the centre of a flashing cross that appears across the screen in different locations.	N/A
Paired Associates Learning (PAL)	The participant is required to learn the spatial location of patterns across a matrix of boxes. The task progresses in stages, from two to eight patterns. If the participant identifies all patterns of a stage correctly they progress to the next stage. If patterns are not correctly identified after 10 trials the task is terminated.	Visual episodic memory; learning
Delayed Matching to Sample (DMS)	A “sample” pattern is presented, followed by four similar “choice” patterns. The participant is instructed to identify the choice pattern that matches the sample. Choice patterns are presented simultaneous to the sample, or after a delay of 0, 4 or 12 s.	Simultaneous visual pattern recognition; short-term visual memory
Verbal Recognition Memory (VRM)	A list of 12 words is presented in succession and the participant is instructed to read each word aloud. The participant is then asked to recall as many words as possible, and distinguish between words from the original list and distractor words.	Verbal memory
Reaction Time (RTI)	A yellow spot appears on the screen and the participant is instructed to touch the spot as fast as possible. In the “Simple” stage, the spot will appear in only one location for 10 trials. In the “Choice” stage, the spot will appear in one of five locations for 15 trials.	Simple and choice reaction time; processing speed
Rapid Visual Information Processing (RVIP)	The digits 2 through 9 are presented at a rate of 100 digits per minute. Participants are instructed to detect three target sequences of digits (3-5-7, 2-4-6 and 4-6-8) and to register their response using a press pad.	Visual sustained attention; processing speed
Spatial Working Memory (SWM)	Coloured boxes are presented on the screen. The participant is instructed to find a blue token in each of the boxes using a process of elimination. The task progresses from 3 to 8 boxes.	Spatial memory; spatial working memory; heuristic strategy; executive function
One Touch Stockings of Cambridge (OTS)	The screen is divided into two halves, each of which contains a display of three coloured balls arranged across three “stockings”. The participants is asked to determine the minimum number of moves required to match the position of balls in lower display to the upper display, moving only one ball at a time.	Spatial planning, spatial working memory, executive function
Attention Switching Task (AST)	Arrows appear on each side of the screen and the participant is cued to indicate the direction the arrow is pointing, or the side of the screen on which the arrow appears using a press pad.	Attentional set-shifting; processing speed; executive function
